# Are Canadian medicine librarians directly supporting medical student health and wellness? A nation-wide survey

**DOI:** 10.29173/jchla29565

**Published:** 2021-12-01

**Authors:** Jackie Phinney, Lucy Kiester

**Affiliations:** 1 Instruction/Liaison Librarian: Dalhousie Medicine New Brunswick, Dalhousie University, Saint John, NB; 2 UGME Liaison Librarian, McGill University, Montreal, QC

## Abstract

**Introduction:**

Students in Undergraduate Medical Education (UGME/UME) programs face a variety of stressors that can affect well-being. To address this, the Committee on Accreditation of Canadian Medical Schools (CACMS) mandates that medical schools offer support and programming that promotes student well-being. Academic librarians are accustomed to providing outreach that meets their faculties’ needs. Therefore, the goal of this study was to explore if Canadian undergraduate medical education librarians are supporting medical student wellness at their medical schools, and how.

**Methods:**

A bilingual, electronic survey containing multiple choice and open-ended questions was distributed across two Canadian health sciences library listservs during the summer of 2020. Librarians supporting UGME/UME programs now or within the last three years were invited to participate.

**Results:**

22 Responses were received, and 17 complete datasets were included in the final results. The majority of respondents have encountered a medical student in distress (n=10) and have adjusted their teaching style or materials to help reduce stress in medical students (n=9). Other initiatives such as resource purchasing, wellness-themed displays, planning wellness-themed events and spaces, and partnerships on campus in support of medical student wellness were less common.

**Discussion:**

The data in this study provides evidence that Canadian undergraduate medical education librarians are mindful of medical student well-being, and are taking steps to provide relevant support to this learner group. Librarians could adopt similar initiatives at their libraries to show support for learner wellness, and enhance their programs’ accreditation efforts in this area.

## Introduction

Academic librarians encounter students at numerous points in their educational journey. Due to the ongoing demands of course-work, career planning, as well as personal matters, students in higher-education programs experience highs and lows that librarians may sometimes witness in their supportive roles. The Canadian literature highlights the severity of medical student stress across provinces, as Maser et al noted in their recent paper from 2019 that medical students across Canada had “significantly higher rates of psychological distress, suicidal ideation, and mood and anxiety disorders” when compared with postsecondary graduates from the general population [1]. A 2021 study by Neufeld and Malin looked at the coping strategies used by medical students in Saskatchewan in response to stress, and their findings revealed that third year medical students reported “the most use of denial to cope than all other years” [2]. A study by Matheson et al. examined the sources of stress and support in a group of undergraduate and graduate medical trainees at Dalhousie University, and found that “psychological distress, often in the form of anxiety and depression, is a common experience for medical trainees”. The authors also state that medical schools should “act as key partners in supporting student well-being by promoting self-care...and developing programs to support at-risk students” [3].

A key partner in the delivery of medical education in Canada is the Committee on Accreditation of Canadian Medical Schools (CACMS), which dictates the standards of Canada’s seventeen medical schools. CACMS publishes a handbook of standards that provide “the basis by which the quality of Canadian medical education programs leading to the M.D. degree will be judged in the peer-review process of accreditation” [4]. While library services and the role of professional library staff are accounted for in standard 5 of the 2021-2022 handbook, standard 12 pertains to “medical student health services, personal counseling, and financial aid services” and standard 12.3 in particular outlines that a medical school must have “an effective system of personal counseling for its medical students that includes programs to promote their well-being and to facilitate their adjustment to the physical and emotional demands of medical education” [5]. While providing personal counseling to students is outside the scope of professional librarians, this standard raises the question of how the library can support these efforts to promote physical and emotional well-being in medical students, and if, by doing so, they can contribute to their medical school’s efforts to meet this standard.

The literature provides examples of academic libraries supporting student wellness through various programming efforts. Kohout-Tailor and Klar offered assorted de-stressing activities within their academic library for 2-hours each weekday during an initial exam period, and in subsequent semesters they partnered with staff from student support services and marketed the program campus-wide using promotional materials (including social media) [6]. Walton summarized the responses to an email discussion amongst university libraries in the United Kingdom, where listserv members were asked how they are supporting student well-being at their library. Their responses included providing a physical space where students can have quiet time and relax, a digital library that provides materials to support student well-being, collections of Reading Well books, and other services such as animal visits, gaming sessions, and more [7]. A very recent paper by Bladek outlined various wellness initiatives that have happened at academic libraries, and discussed the literature demonstrating that “academic libraries have actively sought to join larger institutional initiatives or partner with campus units already offering well-being services and resources”[8].

There are countless other examples of fostering student wellness in academic libraries, but the peer-reviewed literature on medical student wellness promotion in libraries is sparse. Herron discusses the use of mobile wellness resources to promote medical student wellness, and the opportunity libraries have to champion for the use of these tools to enhance medical student wellness [9]. Norton provides an example of a therapy dog program at Yale University’s medical library, where healthcare professionals and medical students alike visited with a therapy dog to promote their well-being [10]. These papers, along with examples of library initiatives such as subject guides focusing on medical student wellness [11] demonstrate that some efforts are being made to support medical student wellness within academic libraries.

Placing this topic within the Canadian context, in 2018 through 2019 we conducted an environmental scan of the social media accounts of the Canadian university libraries that support UGME/UME programs [12]. We found that services, resources, and events that support overall student wellness were occurring, but it was unclear if these events involved the librarian(s) who support medicine, or if these events were being directly promoted to medical students. The interest in learning more about direct promotion to students is fueled, in part, by previous work in the academic medical librarianship literature that is unrelated to student wellness, but still noteworthy. In their case report, Vela discusses the use of various communicative methods at their library, and their decision to incorporate a more direct method of reaching medical students to encourage more meaningful engagement [13]. When considering the promotion of student wellness, we wondered if Canadian UGME/UME librarians are making use of direct methods of contact to reach the medical students in support of their wellness, not just for curriculum-related matters. Having direct communication channels between librarians and medical students can increase the likelihood that students will engage with the librarian on a deeper level, possibly leading to a deeper understanding of wellness resources.

Therefore, the purpose of this study was to explore if Canadian Undergraduate Medical Education librarians are supporting medical student wellness, and if so, how. This was accomplished by means of a direct survey. As a secondary question, we were also interested in knowing if librarians are using a direct line of contact to medical students to promote these efforts. Our goal is to shed light on the strategies librarians are using to support medical student wellness in Canada, which could provide insight on additional ways librarians can support this group on a personal level, as well as enhance the accreditation efforts at their respective medical schools. This study was approved by the Research Ethics Boards at Dalhousie University and McGill University, and funding was provided by the Council of Atlantic University Libraries’ Collaborative Research Grant.

## Methods

To accomplish our study goals, we designed a survey that contained both multiple choice and open-ended questions [see Online Supplement 1, Appendix B for the full survey]. This survey was developed with our previous environmental scan in mind, and the questions were inspired by the instances of services, resources, and events we collected [12]. Key concepts (i.e. ‘academic liaison librarian’, ‘self-care activities’, and ‘direct promotion’) were defined within the survey instrument, to ensure a consistent interpretation across participants (See Online Supplement 1, Appendix B for the survey containing these definitions). After confirming that they met the inclusion criteria, respondents were asked if they have encountered medical students who were struggling with their mental health (and if so, how did they address it); if they have ever planned wellness-related events or spaces; if they have purchased wellness-related material for the library; adjusted their teaching style or tools to help medical students dealing with stress, and more. Our participant sample may have included librarians from the two universities in Canada that offer three-year undergraduate medical degrees, as we believed they had valuable data to contribute on this topic regardless of the differences in their program timeline. Therefore, they were also invited to participate anonymously in this study.

Online Supplement 1

After designing the survey, the draft version was piloted amongst a group of academic librarians and medical student affairs professionals. Once the pilot was complete, the finalized survey was translated to French by a translation service at McGill University. Both the English and French versions were then uploaded into the REDCap software for online data collection. Using the listservs for the Canadian Health Libraries Association as well as the Canadian Association of Medical Education Librarians, data collection took place during July and August of 2020 in the midst of the COVID-19 pandemic. All recipients of the recruitment email were invited to click a link to begin the survey, and they then reviewed an informed consent document before proceeding to the survey (see Online Supplement 1, Appendix A for the informed consent document). Participants were free to withdraw at any time by closing their web browser, and incomplete surveys were not included in the final data analysis. Participants were not required to answer all survey questions in order to proceed through the survey.

Upon survey completion, the anonymous data was exported from REDCap into Microsoft Excel for further analysis. Data from incomplete surveys where respondents did not meet the inclusion criteria in question one or did not click the final submit button was excluded at this stage, and the remaining data was cleaned for further interpretation. Respondents were not required to answer every question to proceed to the next, except for the qualifying question at the beginning of the survey. The categorical data was tabulated to provide a clearer indication of results (see Online Supplement 2, Table 1). The answers to open-ended questions were separated and reviewed independently, and are presented in Online Supplement 1, Appendix C in their unedited format and original language, so as to preserve the unique perspectives of the respondents. We enlisted the help of Dalhousie University’s Statistical Consulting Service who performed three Pearson’s chi-square tests on categorical data to determine relationships between specific variables:

1) If respondents answered ‘yes’ to encountering a struggling medical student, were they more likely to answer ‘yes’ to changing their teaching style or materials;
2) If respondents answered ‘yes’ to creating wellness-related displays, were they more likely to answer ‘yes’ to purchasing or recommending wellness-related materials, and
3) If respondents answered ‘yes’ to having partnerships with other units, faculties, or departments in support of medical student wellness, were they more likely to answer ‘yes’ to being involved in the creation of a wellness-related space. The answers to the open-ended questions were not coded to preserve the participants’ voice and keep the context of their comments intact.

Online Supplement 2

## Results

Upon receiving the recruitment email and beginning the survey, participants were asked to indicate if they were currently working as a liaison librarian for an undergraduate medical education program in Canada, or had done so within the last three years. Twenty-two respondents answered yes to this and began the survey, but only 17 were completed and included in the final analysis. As per the survey introduction, only responses that completed the survey were included (See Online Supplement 1, Appendix A; voluntary participation). While none of the questions were mandatory (all except the introductory question were optional), no questions were skipped by any respondent who completed the survey through to final submission.

When librarians were asked if they have ever encountered a medical student who was struggling with their mental health, physical health, or overall wellness, 10 out of 17 respondents indicated that they had. However, only half of those respondents (n=5) indicated that the student(s) disclosed their struggle directly to the librarian. When respondents were asked to provide detail on if they addressed the situation and if so, how, respondents gave insightful answers that demonstrated their ability to take action in these situations (see Online Supplement 1, Appendix C, Item 1).

Turning to purchasing practices in support of medical student wellness, we asked participants if they have ever purchased or recommended library materials that would help students maintain their own health and overall wellness; 8 out of 17 respondents said that they had. When the 8 respondents were then asked to indicate what they had purchased or recommended by checking all that applied, varying materials were selected (see Online Supplement 2, Table 1) with the answers to ‘Other’ being “puzzles, craft supplies, origami paper”, and more (See Online Supplement 1, Appendix C, Item 2). Of the 8 respondents who had purchased or recommended materials, only 2 indicated that these materials had been directly promoted to the medical students.

In relation to purchasing practices, we were also interested to know if our study participants had ever created a display of books or other materials in the library, at the medical school, or virtually, that focused on student health and overall wellness. Most respondents (11 out of 17) had not done so, but the 6 who had indicated that the display was at the health sciences library (n=4), or other (n=2). Those who indicated ‘other’ provided further details on this (See Online Supplement 1, Appendix C, Item 5). Of the 6 respondents who had created a display, most (5 out of 6) noted that it had been directly promoted to the medical students.

Diving deeper into librarian involvement with wellness initiatives in their physical spaces, we asked respondents to indicate if they had ever been involved in the creation of a physical space on campus where students could engage in self-care activities. A majority indicated that they had not been involved in such an endeavour (12 out of 17), but of the 5 who indicated they had been, 4 of those respondents indicated that this space had been directly promoted to medical students. When asked how they were involved in creating a space, the 5 respondents shared that they had created a green space using seeds that students planted, placed white noise machines in the library, and more (see Online Supplement 1, Appendix C, Item 3). Similar to creating a physical space, our survey asked librarians if they had ever planned or been part of planning a health or wellness-related event at their library. Only 6 out of 17 respondents had done so, and when asked to provide more information they reported on a variety of initiatives (See Online Supplement 1, Appendix C, Item 4). Within those 6 respondents, only 2 indicated that the event(s) were directly promoted to medical students.

Exploring the idea of librarian-faculty partnerships, we asked participants to share if they have ever officially partnered with another unit, faculty, or department at their medical school to provide a service, resource, or event that supports medical student health and overall wellness. Only 2 out of 15 respondents answered ‘Yes’ to this question, and when asked who initiated this partnership, these respondents chose ‘other’ and shared that it was “student wellness services (Student Union and Dean of Students)” and a “Healthy Working Group” that initiated the partnerships.

Librarians were asked if they have ever adjusted their own teaching style or teaching materials (including online tools e.g. LibGuides, etc.) in order to help reduce stress in medical students. More than half of the respondents (9 out of 17) replied ‘Yes’ to this, and when asked for more details, they provided a number of examples of this (see Online Supplement 1, Appendix C, Item 7).

Finally, participants were asked to share any final thoughts or examples they had supporting medical student health and overall wellness, and a variety of comments were provided such as: “we do our best to create a welcoming environment”; “one thing I didn't anticipate before becoming a liaison librarian is that we are sometimes a friendly neutral presence”, and more (See Online Supplement 1, Appendix C, Item 8). One participant indicated in their final thoughts that they are not the liaison librarian for medicine. After careful consideration of the definition of ‘academic liaison librarian’ that was included in the survey, we chose to include their data.

In addition to the results stated above, we pursued further analysis to answer three specific questions listed in the methods section that would explore relationships between variables. It was concluded that these tests were all insignificant, possibly due to the low power from our small sample size.

## Discussion

The purpose of this study was to determine if librarians who work with undergraduate medical students in Canada are supporting their wellness. We were also interested in understanding if librarians were utilizing direct promotion methods to share their wellness initiatives with medical students, and we explored these questions through a survey containing both multiple choice and open-ended questions. Upon consideration of the data, it is evident that it can be organized into two predominant themes:

### Librarian Awareness of Student Needs

Out of 17 respondents, 14 indicated that they have been engaged with wellness activities in some way, to greater or lesser extent depending on the respondent and the activity. Overall, there is clearly an awareness in the library profession and within health libraries that wellness is an issue when working with UGME/UME students.

Nearly half of respondents (8 out of 17) noted that they had purchased or recommended library materials that would help students maintain their overall health and wellness, while only 6 out of 17 respondents reported that they created displays involving wellness-related materials. It is possible that this discrepancy is due to different operating processes of different libraries, wherein library non-academic staff may be in charge of item displays. Some of the respondents who did not purchase or recommend materials may simply have no collections duties; we cannot be sure.

Over half of the respondents (n=9) had made teaching modifications in response to student wellness needs. These modifications came in many forms; some of the interesting ones include highlighting mental health apps in an App Guide, drop-in support sessions to be as accommodating to student needs as possible, or simplifying instructions and walking students through activities step-by-step to help alleviate mental load. This awareness of alleviating the mental loads placed on medical students is supported by Slavin’s commentary on the work that the Saint Louis University School of Medicine did, whereby the reduction of students’ cognitive load contributed to “striking decreases in adverse mental health outcomes” over a ten-year period [14]. Modification of librarians’ teaching personas in response to different contexts has been discussed in the literature by Azadbakht, who conducted a survey and found that academic librarians “viewed themselves as being highly adaptable and responsive to students’ needs” [15]. We believe the findings from our study can help ignite the discussion on how librarians can support medical student wellness through pedagogical methods.

The highest number of responses to a scenario we presented came when we asked if respondents had encountered a student who is having mental health difficulties, with 10 individuals saying that they had had such an encounter. Responses to these encounters were highly varied. For those who took action, interventions included referral to services, listening while a student expressed themselves, and a modification of an assignment with additional support. Similar to listening, one respondent described offering support to a crying student in a washroom, however the student had declined to reach back out to them at that time. Only one respondent took no action, and we cannot know why they chose not to intervene, or if by no action they simply meant that they offered a sympathetic ear.

Of the 7 respondents who had not encountered a student in distress, three indicated some other involvement in student wellness, be it in purchasing, modifying teaching, or some combination thereof; showing that even if a respondent hadn’t had a personal interaction with a distressed student, that does not preclude them from being aware of potential wellness resources or needs.

Our survey had a number of respondents who answered no to all our questions that we identified as being central to identifying or addressing student wellness needs (see Online Supplement 1, Appendix B; questions 1, 2, 5, and 7). Three respondents answered no to all of these questions, with an additional respondent who answered no to the three questions (see Online Supplement 1, Appendix B; questions 1, 2, and 7) discussed above, but who had been involved in the creation and promotion of a wellness book display. It would be interesting to gather further data from these respondents on why they have not engaged in any of these actions, as our survey provides us with no way to know if this is by choice, by necessity, or by a restriction of some other kind. Interestingly, Bladek [8] notes that important questions remain such as, “are there particular professional competencies that academic librarians need to develop in order to support student well-being”? Bladek’s statement causes us to question if the respondents who answered no to these questions are uncomfortable supporting wellness due to their lack of confidence in this area, or if they are simply lacking the resources to do so.

### Engagement and Promotion Efforts

It is clear that most respondents are aware of wellness-related issues with medical students (10 out of 17), and it is positive to see that half of the respondents who have encountered a student in distress (n=5) have taken steps to help in the moment of encounter. However, in reviewing the data on librarians’ involvement in purchasing or recommending wellness-related library materials, creating wellness-themed displays in the physical or virtual space, creating a wellness-related physical space on campus, participating in the running of a wellness-related event at the library, or partnering with another unit on campus to provide a wellness-themed service, resource, or event for medical students, most respondents answered ‘No’ to involvement in these activity types. Therefore, if a majority of respondents have encountered these students in some sort of wellness crisis, and 9 out of 17 of them have even taken the steps to modify their own teaching style or materials to better support student wellness, why aren’t other initiatives happening at a rate that matches the librarians’ awareness?

With a small dataset that does not provide direct insight into each respondents’ situation, it is difficult to determine the exact reason for this. Some potential reasons could be lack of spaces or resources (physical and/or human) to make these initiatives happen. It is also possible that other librarians, library staff, or separate branches are engaging in these activities and the respondent was unaware of this at the time of survey completion. Financially, there could also be budget limitations within the library that make it difficult to purchase materials that are outside the core titles needed for curriculum support, or plan events that require supplies. From a workload perspective, the respondents may already be overwhelmed with their own daily tasks, and while having a momentary encounter with a student in need or making intentional tweaks to teaching materials is feasible, going beyond that may be unmanageable.

Somewhat disappointing was the finding that 15 out of 17 respondents have not partnered with another unit, faculty, or department at their medical school to provide a service, resource, or event that supports medical student health and overall wellness. However, those who reported having these partnerships (n=2) indicated that they were contacted by other campus organizations to participate in such an event. This is further complemented by the fact that those respondents who had existing partnerships (n=2) were included in the set of respondents (n=5) who indicated that they had referred a student in distress to outside resources in question 1.A of the survey (see Online Supplement 1, Appendix B, Section B). The idea of campus partnerships in support of medical student wellness is an important area for undergraduate medical librarians to consider, and collaborative partnerships can help the academic library “have a greater impact in helping students learn how to care for their well-being during stressful times” [6]. Given the librarians’ awareness of student experiences in times of stress, tapping into existing networks on campus, or creating new ones, could help the library support UGME/UME’s mandate to provide “programs to promote [student] well-being and to facilitate their adjustment to the physical and emotional demands of medical education” [5]. The idea of partnering with others to deliver services is strengthened by Matheson et al.'s findings which suggest that “if interventions are to be effective, they should be tailored to the students’ needs commensurate with their particular level of educational programming” [3]. The groups who are already working with medical learners likely have a firm understanding of how best to reach them, and libraries could benefit from that knowledge-sharing.

A secondary question in this study was to determine not only if librarians are supporting medical student wellness, but to see if they are directly promoting these efforts to medical students. In keeping our previous social media scan in mind [12], we asked respondents a number of questions to determine if they had supported wellness-related efforts that could be characterized as services, resources, or events for the undergraduate medical students at their campus. If a respondent answered yes to four of these questions in particular, we asked a follow-up question on if medical students received any direct promotion as defined in the survey instrument. In asking this follow-up question, our goal was to explore if the library has direct channels of contact with the medical students, which was something we could not determine in our past social media scan due to feasibility and online privacy restrictions (i.e. closed social media groups, private listservs, etc).

Interestingly, the respondents indicated that if they had participated in the creation of a physical space on campus to partake in wellness activities, or if they had created a wellness-related display in the physical or virtual space, it was more likely to be directly promoted to medical students. However, when asked if they had purchased or recommended library materials related to wellness, or if they had been involved in planning a wellness-related event, those who answered yes mostly indicated that these were not directly promoted to medical students (see Online Supplement 2, Table 1).

The finding that events were not directly promoted to medical students is interesting given that events would, theoretically, be more promoted given the time sensitive nature of them. However, it is possible that, as we surveyed only librarians, promotion of events was handled by another department or staff member and the librarian was unaware of these efforts. When considering the finding that purchased library materials related to wellness were not highly promoted to the medical students directly, this could be due to a number of factors including a delay in purchase requests and items coming in, the workflow involved in processing them, or just a concern that promoting a single title could lead to over demand.

Somewhat contradictory to this is the data showing that wellness-related displays were directly promoted to the medical students. One theory for this is that the librarians who have created displays are not promoting just their own purchases, but materials throughout the collection that other library staff have purchased. Finally, it was interesting that the physical space on campus to partake in wellness activities was directly promoted to students, and without having more context on the processes involved in creating these spaces or the people who were involved in creating them, conclusions cannot be drawn.

It is therefore difficult to determine the applicability of these findings to the wider undergraduate medical librarian population due to the very small subset of respondents who answered these secondary questions, and without knowing the individual practices at the respective libraries we can only appreciate the findings at face value. As Vela notes, “finding a reliable way to connect with their student base is a challenging task for any librarian”, and “the best and most effective method will vary depending on each student population” [13]. Further studies on communication practices in librarianship could explore how librarians are directly promoting their wellness-related events, services, and spaces to medical students, but should take into account the unique factors at each individual library.

### Responses Related to COVID-19

In addition to the two major themes above, some respondents opted to include commentary on how the ongoing COVID-19 pandemic has affected their involvement with student wellness. While we were unable to change our survey questions to address the current events at the time of data collection, we were intrigued to receive some responses that highlighted how the pandemic, wellness, and libraries were interacting. We feel it is important to highlight these responses here.

One respondent stated the following in regards to their own teaching: “Avec la situation actuelle (Covid), réduire le contenu des formations [...] essayer de fournir le plus possible les manuels en ressources électroniques accès illimité afin de réduire le stress financier” (i.e. they had worked to reduce content, and were pushing library resources to help those who might be experiencing financial difficulties due to the pandemic). Another respondent discussed how events planned for wellness had to be cancelled due to COVID Restrictions, and an additional response highlighted that their promotion of LibGuide displays of titles was “enhanced significantly in the last year and very much so since COVID-19.”

Many respondents did not address their situation in light of the COVID-19 pandemic. However, seeing as there were no explicit questions pertaining to the pandemic, this is perhaps not surprising. It was reassuring to see that the respondents who did address this were concerned with doing what they could to continue promoting wellness in some way, even during a global crisis. It would be interesting in future studies to evaluate if wellness support at medical campus libraries grew or diminished during the pandemic, or to investigate physician burnout and wellness work related to it. Due to the continuity of the current pandemic it would be premature to ask those questions at this time.

## Limitations and Further Study Recommendations

The small sample size was a limitation we were aware of going into this project, as we specifically wanted to target UGME/UME-serving librarians within Canada’s seventeen medical schools. Future studies could explore this topic further with a larger sample size that includes librarians who support different health sciences programs. In planning this study, we did not anticipate that data collection would take place during a global pandemic. This timing may have impacted the richness of our data (due to there being a lack of recent campus services/events for our sample to report on), as well as the likelihood of the target population taking the time to respond when already feeling stressed due to increased workloads, uncertainty, and family demands. We recognize the irony in that our study sought to examine wellness-related initiatives targeted at medical students, at a time when academic staff wellness was heavily impacted by an ongoing pandemic.

As with any survey, we were limited by several possibly subjective terms. We did provide our own definitions for terms such as “self-care”, “direct promotion”, and “liaison librarian,” however it is possible that these terms were interpreted differently by participants, which could affect who participated in the survey, as well as how those who did participate chose to interpret and respond to questions. For example, we defined UGME/UME “liaison” as anyone who engaged with the undergraduate medical school as part of their role as librarian; this was purposefully left broad to try and include possible collections librarians, or those who don’t primarily liaise with the medical school but may help provide instruction. However, it is possible that participants saw the title and self-selected out of participating prior to reading our definition. In regards to the subjective term of “wellness,” in their paper outlining issues that need to be addressed to make student well-being services sustainable in academic libraries, Cox and Brewster note that papers describing well-being interventions at libraries “fail to convincingly define the core concept of well-being that the activities they are organising are trying to address”[16]. Therefore, it is possible that our survey respondents held differing views of what a wellness initiative is, and responded accordingly.

Further directions of similar studies could include expanding this topic into interviews or focus groups. Given how frequently we received long text-based answers (see Online Supplement 1, Appendix C), it could be that we would have a good response rate for such a call. These interviews would allow us to elicit further details from participants on wellness support for UGME/UME students specifically. Another possible question could be how do librarians promote or reach out directly to medical students? It is clearly something that some librarians are doing, while others are not; inquiring further into how this is done, or why it is not done, would be an interesting topic to explore further.

Lastly, it is important to be mindful that interventions at the library can help mitigate the pressures faced by medical students, but if students face serious mental health issues they require interventions that are beyond the scope of libraries. While the topic of library support for medical student wellness is an important one, we must acknowledge our professional limitations and recognize there is only so much that can be done within the scope of librarianship. In line with Cox and Brewster’s insight into the problems that arise with libraries’ supporting student well-being “without some expertise on what well-being means and how to address it, initiatives are unlikely to have great plausibility” [16]. It is possible that medical librarians feel unqualified to support wellness initiatives, and further research could explore librarians’ willingness and comfort in supporting the well-being of medical students.

## Conclusion

Undergraduate medical education librarians in Canada care about medical student well-being, and they are demonstrating this through various initiatives outlined in this research such as planned spaces, student-centered teaching, thoughtful collections purchases, and more. The results from this survey provide some evidence as to ways that librarians, both in medical education and other disciplines, can support their students’ health and wellness. Medical librarians could work with faculty or other partners to support initiatives for these undergraduate students who are at a high risk of burning out. All of these efforts — collections, teaching, partnerships etc. —could provide further evidence for how the library can contribute to accreditation standards that are outside the traditional library domain.

## References

[1] Maser B, Danilewitz M, Guérin E, Findlay L, Frank E. Medical student psychological distress and mental illness relative to the general population: A Canadian cross-sectional survey. Acad Med. 2019;94 (11):1781-1791. doi: 10.1097/ACM.0000000000002958 31436626

[2] Neufeld A, Malin G. How medical students cope with stress: a cross-sectional look at strategies and their sociodemographic antecedents. BMC Med Educ. 2021;21(1):299. doi: 10.1186/s12909-021-02734-4 34034732 PMC8152145

[3] Matheson KM, Barrett T, Landine J, McLuckie A, Soh NL, Walter G. Experiences of psychological distress and sources of stress and support during medical training: A survey of medical students. Acad Psychiatry. 2016;40(1):63-68. doi: 10.1007/s40596-015-0395-9 26223316

[4] Committee on Accreditation of Canadian Medical Schools (CACMS). Standards and Elements [Internet]. [Cited 2021 Jan 8]. Available from: https://cacmscafmc.ca/accreditation-documents/2021-2022/standards-and-elements

[5] CACMS Standards and Elements | CACMS [Internet]. [cited 2021 Jan 8]. Available from: https://cacmscafmc.ca/sites/default/files/documents/CACMS_Standards_and_Elements_AY_2021-2022.pdf

[6] Kohout-Tailor J, Klar CL. Growing collaborative outreach efforts to support the well-being of communities. JLOE. 2020 Oct 26;1(1):6–12. doi: 10.21900/j.jloe.v1i1.463

[7] Walton G. Supporting student wellbeing in the university library: A core service or a distraction? New Rev Acad Librarian. 2018 Apr 3;24(2):121–3. doi: 10.1080/13614533.2017.1418240

[8] Bladek M. Student well-being matters: Academic library support for the whole student. J Acad Libr. 2021;47(3):N.PAG. doi: 10.1016/j.acalib.2021.102349

[9] Herron J. Mobile wellness resources. J Electron Resour Med Libr. 2016 Jul 2;13(3):94–8. doi: 10.1080/15424065.2016.1225542

[10] Norton MJ, Funaro MC, Rojiani R. Improving healthcare professionals’ well-being through the use of therapy dogs. J Hosp Librarian. 2018 Jul 3;18(3):203–9. doi: 10.1080/15323269.2018.1471898

[11] Epstein S. Subject Guides: Mental Health and Wellness: Mental Health Resources [Internet]. [cited 2021 Jan 8]. Available from: https://medfsu.libguides.com/mentalhealthforproviders/mentalhealth

[12] Phinney J, Kiester L. Are university libraries supporting medical student wellness? Results from an exploration of library social media. 2019 [cited 2021 Jan 8]; Available from: https://DalSpace.library.dal.ca//handle/10222/76229

[13] Vela K. Using slack to communicate with medical students. J Med Libr Assoc. 2018 Oct; 106(4): 504-507. doi: https://dx.doi.org/10.5195%2Fjmla.2018.482 30271297 10.5195/jmla.2018.482 PMC6148623

[14] Slavin S. Reflections on a decade leading a medical student well-being initiative. Acad Med. 2019 Jun;94(6):771-774. doi: 10.1097/ACM.0000000000002540 30489287

[15] Azadbakht ES. The many faces of instruction: An exploration of academic librarians’ teaching personas. Comm Info Lit. 2021;15(1):57-74. doi: 10.15760/comminfolit.2021.15.1.3

[16] Cox AM, Brewster L. Services for student well-being in academic libraries: Three challenges. New Rev Acad Libr. 2021 27:2, 149-164. doi: 10.1080/13614533.2019.1678493

