# Supplementary material for: Are Canadian medicine librarians directly supporting medical student health and wellness? A nation-wide survey
Source: J Can Health Libr Assoc. 2021 Dec 1;42(3):164–73. doi: 10.29173/jchla29565 (PMC9327600; doi:10.29173/jchla29565)
Supplement: Supplementary file 1 — Online Supplement 1 [file JCHLA-42-164-s001.pdf]

Appendix A: Informed Consent Form (English version)

Study Name: Are Canadian medicine librarians directly supporting medical student health and wellness? A nation-wide survey

Researchers: Jackie Phinney, Instruction/Liaison Librarian, Dalhousie Medicine New Brunswick of Dalhousie University; Lucy Kiester, Assistant Librarian UGME, McGill University

Purpose of the Research: The purpose of this research is to determine if undergraduate medical education librarians in Canada are supporting medical student wellness, as well as if these efforts are being promoted directly to students. The findings will contribute to original research in this area and inform the health sciences library community of ways they can engage with student wellness.

Who Can Take Part in This Study: Librarians who are currently working with undergraduate medical education in some capacity, or have in the last 3 years.

What You Will Be Asked to Do: You are being asked to fill out an online questionnaire, which will take approximately 10 minutes to complete.

Risks and Benefits:The direct risks of this study are minimal. However, some questions in this survey may trigger difficult emotions. If this occurs, you may exit the survey at anytime by closing your browser. We also encourage you to contact your local counseling services should this occur.

There will be no direct benefit to you for participating in this research and you will not receive compensation. However, this research might contribute to new knowledge on how undergraduate medical librarians can support student wellness, which can inform the professional practice of our colleagues nationally and internationally.

Voluntary Participation/Withdrawal from the Study: Your participation in the study is completely voluntary and you may choose to stop participating at any time. Responses are anonymous, therefore once the final responses have been submitted they cannot be removed from the data set. Data from incomplete surveys will not be included in the final analysis.

Confidentiality: The survey is anonymous and no identifying information will be collected. Survey data will be collected through the Dalhousie University secure server using REDCap survey software and will be stored in a password-protected account. Researchers will keep all your survey responses confidential, survey completion is anonymous, and there will be no link to your IP address or any personal identifiers. Individual quotations may be used in the public presentation of results at professional conferences and/or journal publications; however, quotations will not be linked to individuals. Once the study is complete, anonymous data will remain on a password-protected computer. There are no plans to destroy data.

Obtaining results: We will provide you with a short description of group results when the study is finished, if desired. No individual results will be provided. You can request these results by emailing either researcher at the contact information listed below.

Questions about the Research? If you have questions about the research in general or about your role in the study, please feel free to contact Jackie Phinney (j.phinney@dal.ca) or Lucy Kiester (lucy.kiester@mcgill.ca) by email. The plan for this study has been reviewed for its adherence to ethical guidelines and approved by the Research Ethics Boards at Dalhousie University [File # xxxxx] and McGill University [File # xxxxx].

We thank you for your time!

By clicking on the 'Next' button below, I agree to participate in this survey. I am aware that I am free to withdraw from this survey at any time up until I submit my responses to the survey.

[Next Button]

Appendix B: Survey (English version)

Section A: Introductory Question

Are you or have you been an academic liaison librarian* for a 4-year undergraduate medical program in Canada, either now or within the last 3 years?

*Academic liaison librarian= For the purposes of this survey, an academic liaison librarian is someone who supports or engages with the undergraduate medical school as part of their librarian role

a) YES Proceed to question 1

b) NO Proceed to end of survey

Section B: Survey

1) In your role as a liaison librarian to undergraduate Medicine, have you ever encountered a medical student who was struggling with their own mental health, physical health, or overall wellness?

a) YES Proceed to 1.A.1

1.A.1: Please describe if you addressed the situation and if so, how (eg referred to somewhere, provided phone numbers for help, etc).

____

1.A.2: Did the student disclose their struggles to you directly?

i) Yes

ii)No

b) NO Proceed to question 2

2) In your role as a liaison librarian to undergraduate Medicine, have you ever purchased or recommended library materials that would help students maintain their own health and overall wellness?

a) YES Proceed to 2.A.1 and 2.A.2

2.A.1: What did you purchase or recommend? Please check all that apply:

i) Books (print or electronic)

ii) Mobile apps

iii) Videos

iv) Free materials (print or electronic)

v) Websites (eg mental health websites, time management websites, etc)

vi) Other________________

2.A.2: Were these materials promoted directly* to the medical students?

i) Yes

ii) No

*Direct promotion= targeting medical students specifically through in-person announcement, posters within the medical school, online communication in the form of email to their classes, posting to their learning management system, posting to exclusive social media pages, or other methods of direct communication not listed.

b) NO Proceed to question 3

3) In your role as a liaison librarian to undergraduate Medicine, have you ever been involved with the creation of a physical space on campus where students could engage in self-care activities*?

* self-care activities = relaxation, physical exercise, meditation, sleep, or self-expression such as writing, creating art, or music-making.

a) YES Proceed to question 3.A.1

3.A.1: Please describe how you were involved in creating this space:

____

3.A.2: Was this space promoted directly* to the medical students?

i) Yes

ii) No

*Direct promotion= targeting medical students specifically through in-person announcement, posters within the medical school, online communication in the form of email to their classes, posting to their learning management system, posting to exclusive social media pages, or other methods of direct communication not listed.

b) NO Proceed to question 4

4) In your role as a liaison librarian to undergraduate Medicine, have you ever planned or been part of planning a health or wellness-related event at your library?

a) YES Proceed to 4.A.1

4.A.1: Please describe this event:

____

4.A.2: Was this event promoted directly* to the medical students?

i) Yes

ii) No

*Direct promotion= targeting medical students specifically through in-person announcement, posters within the medical school, online communication in the form of email to

their classes, posting to their learning management system, posting to exclusive social media pages, or other methods of direct communication not listed.

b) NO Proceed to question 5

5) In your role as a liaison librarian to undergraduate Medicine, have you ever created a display of books or other materials in the library, at the medical school, or virtually, that focused on student health and overall wellness?

a) YES Proceed to 5.A.1

5.A.1: Where was this display?

i) Health sciences library

ii) Other library branch

‘ iii) Medical school

iv) Other:________________

5.A.2: Was this display promoted directly* to the medical students?

i) Yes

ii) No

*Direct promotion= targeting medical students specifically through in-person announcement, posters within the medical school, online communication in the form of email to their classes, posting to their learning management system, posting to exclusive social media pages, or other methods of direct communication not listed.

b) NO Proceed to question 6

6) In your role as a liaison librarian to undergraduate Medicine, have you ever officially partnered with another unit, faculty, or department at your medical school to provide a service, resource, or event that supports medical student health and overall wellness?

a) YES Proceed to 6.A.1

6.A.1: Who initiated this partnership?

i) The liaison librarian(s)

ii) The medical school

iii) Somebody else at the library (eg staff, administration, etc)

iv) Other: ____________

b) NO Proceed to question 7

7) In your role as a liaison librarian to undergraduate Medicine, have you ever adjusted your own teaching style or teaching materials (including online tools eg LibGuides, etc.) in order to help reduce stress in medical students?

a) YES (Please explain) ______________________

b) NO Proceed to question 8

8) Do you have any final thoughts or examples to share about your experience supporting (either directly or indirectly) undergraduate medical student health and overall wellness? ______________________________________

Thank you for your time!

Appendix C: Full responses to open-ended questions

1. If participants answered yes to survey question 1 ("In your role as a liaison librarian to undergraduate Medicine, have you ever encountered a medical student who was struggling with their own mental health, physical health, or overall wellness") , participants answered the open-ended question "Please describe if you addressed the situation and if so, how (eg referred to somewhere, provided phone numbers for help, etc)." with the following:
    - Referred & provided phone numbers for Office of Health Profession Student Affairs
    - The student I was working with had some physical health issues causing him to take some time away for the UME program. Because he wanted to fill his time with something useful, he was participating in a systematic review. I helped him to develop his search strategy. He did not share the specifics of his physical health condition and I did not want to pry. It sounded like he was getting the help he needed, so I did not free inclined to offer additional support beyond encouragement for the project he was working on.
    - generally in my experience, the student is explicit about their situation and tells me that they are receiving care/support from various sources. I am then able to continue supporting them in the same ways from my own position (extensions on assignments, more individual time, or similar). i have not experienced being the first line of contact for a student in need of supports.
    - Encountered a young woman crying in the washroom cubicle during exams. I assured her that she wasn't alone and that I was available for her if she wanted to talk. I waited outside for ten minutes, but she didn't come out. About half an hour later, I saw her (recognized the shoes) leave the library in the company of a young man, who was consoling her. Had she asked for help, I could have referred her to several different support resources and walked her to one of them (eg: Student Help).
    - Did not address
    - I've had several students in the past be either overwhelmed with the amount of work they are required to do, or have encountered people in authority positions that have been bullying them or created a toxic environment. I addressed the situation by either referring them to the on campus counselling services or the UGME office for student wellness.
    - Spoke to the student and suggested resources. Provided a sympathetic ear.
    - Assignment deadlines were extended and additional teaching was provided to the students.
    - A few things here. One situation I am thinking of particularly, was one girl who just seemed to need to talk for a while. I would listen, but not provide any advice or anything. She just talked about her life for a while and then would move on. Others, I just see that they seem stressed, or tired. I do not intervene.
    - Ecoute et aiguillage vers des ressources (Translation: Listened and referred to resources)

2. If answered yes to survey question 2 (“In your role as a liaison librarian to undergraduate Medicine, have you ever purchased or recommended library materials that would help students maintain their own health and overall wellness?”), participants were asked “What did you purchase or recommend? Please check all that apply (Other-Please specify)” the responses to “other” were:

- Puzzles, Craft supplies, origami paper, we also have a fiction book collection (which we borrow for a period of time and return - so they do not get entered in the catalogue) but this collection also includes comic books eg some graphic medicine comic books
- “Luminothérapie, zoothérapie, matériel pour se détendre: cahiers colorier, casse-têtes, jeux d'échec en bibliothèque ainsi que collection de bandes dessinées fournie par la bibliothèque et placée dans le local étudiant (rotation des titres mensuellement)”

3. If answered yes to survey question 3 (“In your role as a liaison librarian to undergraduate Medicine, have you ever been involved with the creation of a physical space on campus where students could engage in self-care activities?”), participants answered the open- ended question “Please describe how you were involved in creating this space” with the following:

- In our library, we dedicated an alcove to soft seating, included a "take a book/leave a book" fiction area and a full spectrum lamp. This area is also used by University-level wellness programs, who come in during high stress times (eg: exams) and offer dog therapy, people to talk to, and distractions such as games, puzzles, sometimes take-aways like ear-plugs, fruit, granola bars, tea, etc.
- I gave ideas for activities that could be done in this space, such as workshops, colouring pages, books, origami or puzzles.
- Prior to COVID we were developing a seedling project where we would have two events including planting seeds, having them provide greenery in the space and then members of our university could take them home and grow in their space/garden and have health food/greenery.
  We have also purchased puzzles for the space and provide PR events including Valentine's making, Christmas/holiday card making, hot chocolate and cookies during stressful exam prep etc.
- These are temporary spaces, but we try to make a stress reliever desk in the library for around exam time. We also have placed white noise machines in the quieter areas of the library. We have added plants to improve wellness. So not a space dedicated to wellness, but trying to make our library space more calming.
- Choix du mobilier pour la collection de livres Bien-• être étudiant (et choix de ces livres aussi) qui se trouve dans l'espace détente (jeux d'échecs, coloriage, etc)

4. If answered yes to survey question 4 (“In your role as a liaison librarian to undergraduate Medicine, have you ever planned or been part of planning a health or wellness-related event at your library?”), participants answered the open-ended question “Please describe this event” with the following:

- Every term we have an 'UnStudy' spot where several stations are set up to encourage de-stressing activities. This is in the same section of the library as our BookStop which is our collection of fiction and comic books. These stations include puzzles, colouring, origami, sudoku and word searches. We also have a therapy dog visit the library twice a week leading up to and during exams. At other libraries on campus there has also been escape rooms, yoga/mindful moments, growing edible plants initiatives
- Part of celebration when we installed our variable height desks. Also often take part in therapy dog days, though this and other wellness events are planned and managed by non-academic library staff.
- Event did not take place during COVID. Other PR events (Valentine's making, hot chocolate/cookie events etc) are primarily planned by our AA and in consult with our Director.
- stress reduction activities in the library during exam times - not solely for undergraduate medical students but for all students
- stress reduction tables. Also, we have broadcast the olympics in our computer lab. we encourage our students to create art and display it in the library. We also had therapy dogs for a couple years during exam time, but now we aren't allowed.

5. If answered yes survey question 5 (“In your role as a liaison librarian to undergraduate Medicine, have you ever created a display of books or other materials in the library, at the medical school, or virtually, that focused on student health and overall wellness?”) participants were asked “Where was this display?” The responses to “Other-Please specify” were:

- LibGuide display - promoted in the last 8 years however enhanced significantly in the last year and very much so since COVID-19.
- Dans 5 bibliothèques. Mon collègue bibliothécaire en psycho et moi-même avons soumis un projet pour développer une collection de livres Bien-• être étudiant. Le projet a été reçu avec enthousiasme par la Direction des bibliothèques et on a reçu 5 fois plus d'argent pour acheter 5 copies de chaque livre et placer une collection dans 5 bibliothèques du réseau!

6. If answered yes to survey question 6 (“In your role as a liaison librarian to undergraduate Medicine, have you ever officially partnered with another unit, faculty, or department at your medical school to provide a service, resource, or event that supports medical student health and overall wellness?”) participants were asked “Who initiated this partnership?” The responses to “Other-Please Specify” were:

- Student Wellness services (Student Union and Dean of Students)
- Healthy Workplace Group

7. If answered yes to survey question 7 (“In your role as a liaison librarian to undergraduate Medicine, have you ever adjusted your own teaching style or teaching materials (including online tools eg LibGuides, etc.) in order to help reduce stress in medical students?”) participants were asked to explain their modifications. The responses were:

- Suggestions of mental health apps in App Guide
- eg. provide as many follow-up materials as possible. So if there is an exam coming up the day I am teaching, the materials are there for future reference and can be reviewed at their own pace. Also provide follow-up drop in sessions, where students could meet one on one without having to email to schedule a consultation
- Try to provide teaching material in a variety of media, and in advance of the class wherever possible (or at the beginning of the class itself). We try and coordinate assignment schedules that fit in with the rest of their timelines to reduce stress in that way, and will answer questions in any format the student prefers (drop in, appointment, email, phone, text, virtual meetings). I'm very aware that not all of their learning activities can be flexible, and so I try to do what I can whenever possible.
- I try to incorporate different types of learning materials into my sessions to engage different types of learners in hopes of reducing their stress. Ensuring they feel welcomed to follow up with me.
- A lot of this is just good teaching technique, but:
  1. Anchoring the concepts in prior knowledge, so that you're not asking for a huge cognitive leap. "Remember when you wrote research papers..."
  2. Provide a session outline/outcomes and teach to them without extraneous detail.
  3. Teach practical skills and have the student practice, so that there is immediate application.
  4. Teach at a level where everyone can "get it". Don't overload with extraneous information that librarians love, but no one else cares about. These are smart people, but they get so much shoved at them, their brains can't hold it all, so walk them through it step-by-step.
  5. This isn't a test, it's making sure they have the skill. Make sure they get the skill. Tell them "this isn't a high-stress exercise, if it is high stress, you're doing something wrong, so you should ask"
  6. Make sure they feel safe to ask the question-both in the class and afterwards. I always say "no one is born knowing this stuff" and make sure they know how to find me.
  7. Connect personally with as many of the class as possible - ask names - in the physical class walk around during the assignment time and ask how they are doing.

  Take time to understand their research questions or problems.

  Don't know how this will work online, but demonstrating personal concern for students always pays dividends in the mental health department. Be a real person they can turn to, if they need to.
- Simplified guides and instructions

- I've adjusted my teaching style to be more clear of my expectations for their first year assignment, I have provided links and several points of access where they could reach me. I have also offered flexible hours to meet with the students, depending on their schedule.
- Yes when requested by the School of Medicine, I meet with the student(s) to provide additional training and relax assignment deadlines.
- Avec la situation actuelle (Covid), réduire le contenu des formations... Sinon essayer de fournir le plus possible les manuels en ressources électroniques accès illimité afin de réduire le stress financier

8. The final question ("Do you have any final thoughts or examples to share about your experience supporting (either directly or indirectly) undergraduate medical student health and overall wellness?") was asked of all participants. The responses were:

- I am not the liaison librarian to medicine - I just teach a number of workshops for them. My colleague has done many of the initiatives you listed so far - mental health book collection for example, and the library has hosted zootherapy sessions (where I was not involved in the planning). So although not directly involved with mental health support initiatives, I do address the issue when possible with medical students.
- Our library as a whole has supported students' health and wellness by making puzzles and other calming crafts available, as well as setting up a wellness room during mental health week. I have not been directly involved in those initiatives.
- We do our best to create a welcoming environment. We get resources into their hands with as little fuss as possible. We appreciate that they are working hard in their studies. Having said that, if we offered additional supports to just this one group, do we end up having to provide support for other faculty's learners too?
- I am glad to say that our UGME office and campus student services are very proactive about supporting student health. If they were less visible the library would perhaps step in a bit more; we do provide library space for events frequently, and I try to stay in the loop on the events on campus. One thing I didn't anticipate before becoming a liaison librarian is that we are sometimes a friendly neutral presence; I find I get questions about how the university works, or what's normal or expected in a class or thesis experience by some students who I meet a few times for more traditional reference support, and I try to guide or cheerlead a bit where I can (i.e. confirming when it is time to check with an advisor about something vs. sorting it out on your own). I would say these interactions are more common in grad-level programs and/or with students in international degree streams than with UGME students though.
- I appreciate the materials and training provided by my local institution to recognize students in stress and have used the materials provided to identify these students and refer them to help. I have used it with non-ugme students.
- I sometimes tutor incoming students in the problem based learning small groups. I tell them directly the points on which I am evaluating them, so they can perform to those goals. I always tell my group, specifically, that if they need someone to talk with, I'm there.
