# Supplementary material for: Are Canadian medicine librarians directly supporting medical student health and wellness? A nation-wide survey
Source: J Can Health Libr Assoc. 2021 Dec 1;42(3):164–73. doi: 10.29173/jchla29565 (PMC9327600; doi:10.29173/jchla29565)
Supplement: Supplementary file 2 — Online Supplement 2 [file JCHLA-42-164-s002.pdf]

Table 1: Tabulated responses to multiple choice questions. See Appendix C for responses to the open-ended questions.

In your role as a liaison librarian to undergraduate Medicine, have you ever encountered a medical student who was struggling with their own mental health, physical health, or overall wellness?	Yes(10); No(7)
Did the student disclose their struggles to you directly?	Yes(5); No(5)
In your role as a liaison librarian to undergraduate Medicine, have you ever purchased or recommended library materials that would help students maintain their own health and overall wellness?	Yes(8); No(9)
What did you purchase or recommend? Please check all that apply	Books (Print or electronic) (7); Mobile apps(3); Videos(3); Free materials (print or electronic)(4); Websites (eg mental health websites, time management websites, etc)(4); Other(2)*
Were these materials promoted directly to the medical students?	Yes(2); No(6)
In your role as a liaison librarian to undergraduate Medicine, have you ever been involved with the creation of a physical space on campus where students could engage in self-care activities?	Yes(5); No(12)
Was this space promoted directly to the medical students?	Yes(4); No(1)
In your role as a liaison librarian to undergraduate Medicine, have you ever planned or been part of planning a health or wellness-related event at your library?	Yes(6); No(11)

Was this event promoted directly to the medical students?	Yes(2); No(4)
In your role as a liaison librarian to undergraduate Medicine, have you ever created a display of books or other materials in the library, at the medical school, or virtually, that focused on student health and overall wellness?	Yes(6); No(11)
Where was this display?	Health Sciences Library (4); Other(2)*
Was this display promoted directly to the medical students?	Yes(5); No(1)
In your role as a liaison librarian to undergraduate Medicine, have you ever officially partnered with another unit, faculty, or department at your medical school to provide a service, resource, or event that supports medical student health and overall wellness?	Yes(2); No(15)
Who initiated this partnership?	Other(2)*
In your role as a liaison librarian to undergraduate Medicine, have you ever adjusted your own teaching style or teaching materials (including online tools eg LibGuides, etc.) in order to help reduce stress in medical students?	Yes(9); No(8)
